# Cyclic thrombocytopenia with statistically significant neutrophil oscillations

**DOI:** 10.1002/ccr3.1611

**Published:** 2018-05-31

**Authors:** Gabriel P. Langlois, Donald M. Arnold, Jayson Potts, Brian Leber, David C. Dale, Michael C. Mackey

**Affiliations:** ^1^ Division of Applied Mathematics Brown University Providence RI USA; ^2^ Division of Hematology and Thromboembolism Department of Medicine McMaster University Hamilton ON Canada; ^3^ Department of Medicine General Internal Medicine University of British Columbia Vancouver BC Canada; ^4^ Department of Medicine University of Washington Seattle WA USA; ^5^ Departments of Physiology, Physics, and Mathematics McGill University Montreal QC Canada

**Keywords:** bleeding, cyclic neutropenia, cyclic thrombocytopenia, immune thrombocytopenia, thrombopoietin

## Abstract

Cyclic thrombocytopenia is often misdiagnosed as immune thrombocytopenia due to similar clinical features, a fact of significance because cyclic thrombocytopenia generally responds poorly to treatments used successfully in immune thrombocytopenia. A precise diagnosis must establish the statistical significance of periodicity of the platelet counts using statistical methods (eg, Lomb‐Scargle periodogram).

## 1 INTRODUCTION

Cyclic thrombocytopenia (CT) is a rare blood disorder characterized by periodic cycling in platelet counts. Thrombocytopenia and thrombocytosis can be extreme, resulting in an increased risk of bleeding alternating with an increased risk of thrombosis. The mechanisms that lead to CT may include antiplatelet antibodies, suggesting an autoimmune component with cyclical destruction, or abnormal feedback regulating platelet production or some combination of both mechanisms.^1^ As the natural history of this disorder varies significantly, the relative importance of these mechanisms likely differs across cases. CT is distinct from the more common refractory immune thrombocytopenia (ITP) disorder due to spontaneous undulations in platelet counts. While CT is thought to be rare, it has been observed more frequently in recent years^2^ and may be exacerbated by TPO receptor agonists which are increasingly common in clinical practice.

Despite their differences, in practice CT is often misdiagnosed as ITP because of similar clinical features, which is of significance because CT generally responds poorly to most treatments used successfully in ITP, such as corticosteroids, splenectomy, and intravenous immuglobulin.^1^ Patients with ITP and thrombocytopenia for other causes respond to treatment with thrombopoietin (TPO), but there are few reports of its use in cyclic thrombocytopenia.^3^ To establish a clear diagnosis of CT, therefore, platelet counts must be monitored regularly (3‐4 times weekly for at least 2‐3 weeks), but the precise diagnosis depends on establishing statistical significance of periodicity of the platelet counts using mathematical methods such as Fourier analysis, or more practically the Lomb‐Scargle periodogram.

The mathematical technique of Fourier analysis finds applications to determine periodicities in fields as diverse audio compression in the MP3 format, video compression in JPEG, comparison of DNA and DNA sequence alignment, and cell phone technology. While useful, these techniques rely on sampling data at even periods of time, something both unusual and impractical when counting blood cell numbers. This problem exists in many fields, including astrophysics, in which Lomb^4^ developed an alternative that extends the Fourier analysis method to uneven sampling of data. Scargle^5^ later placed this technique on a solid statistical foundation, resulting in what we call today the Lomb‐Scargle periodogram. This technique is described and used in Fortin and Mackey^6^ in the context of blood cell analysis of patients with periodic chronic myelogenous leukemia, and has been implemented at the website http://cyclicneutropenia.org/ for hematological purposes. It is noteworthy that in a study^7^ of 33 putative cases of cyclical thrombocytopenia, use of the Lomb‐Scargle periodogram analysis identified significant (*P* < .05) periodicity on only 18 instances. Hence, this type of analysis is a valuable adjunct to visual data inspection when trying to distinguish between cases of CT and ITP.

Understanding the clinical and laboratory features of platelet count oscillations, the involvement of other cell lines, and response to TPO stimulation should help clinicians manage this challenging disorder. We describe 2 patients with CT, one of whom was found to have concomitant, statistically significant neutrophil cycling with the same period as the platelets, a feature that has not been previously reported. Both patients received TPO receptor agonists, and treatment was discontinued in both cases because of worsening fluctuations in platelet counts.

## 2 CASE PRESENTATIONS

### 2.1 Patient 1

**Figure 1:**
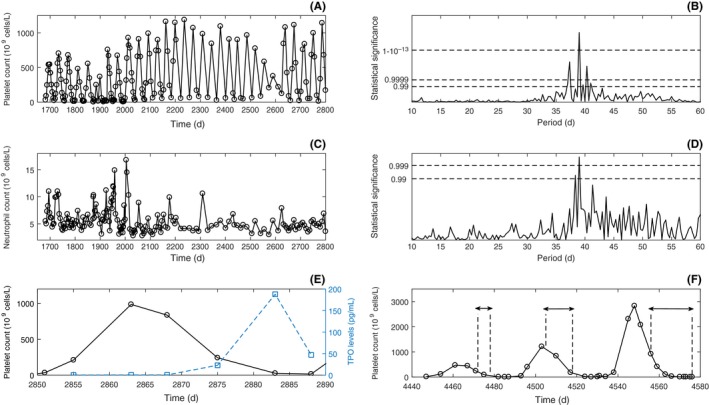
Patient 1 data, with time measured in days since diagnosis. A, Platelet counts (data shown is from April 2003 to April 2006). B, Platelet power spectrum. C, Neutrophil counts (data shown is from April 2003 to April 2006). D, Neutrophil power spectrum. E, Platelet counts and TPO levels over one cycle (data shown is from 23 June 2006 to 30 July 2006, with TPO data points denoted by squares). F, Platelet count for three cycles during three periods of daily treatment with 50 mg of eltrombopag (data shown is from 5 November 2010 to 14 March 2011, with periods of treatment demarcated by dashed lines and double arrows)

A 49‐year‐old male presented to hospital in May 1998 because of spontaneous bruising and mucosal bleeding. His platelet count was 2 × 10^9^/L. The other blood counts were normal, and no other laboratory abnormalities were noted. He had a history of alopecia totalis, but no other concomitant illness and no family history of blood disorders. He was treated with prednisone (100 mg daily) and his platelet count improved, but when the dose of prednisone was gradually reduced and stopped, the thrombocytopenia returned. He subsequently underwent laparoscopic splenectomy in December 1998, which resulted in a positive platelet count response that lasted 4 years. In April 2003, the thrombocytopenia relapsed and after another course of prednisone, platelet count levels displayed a pronounced cyclical pattern of thrombocytopenia alternating with thrombocytosis (Figure 1A), with platelet count fluctuating with a statistically significant (*P* ≤ 10^−22^) period of 39 days (Figure 1B) from nadir values of less than 5 × 10^9^/L to peak values of greater than 900 × 10^9^/L. Statistically significant (*P* ≤ .001, Figure 1D) oscillations of exactly the same period in neutrophil counts (Figure 1C) were also found, but the neutrophil nadir never dropped below the normal range.

Cyclic thrombocytopenia persisted for over 10 years despite treatment with corticosteroids, intravenous immunoglobulin, danazol, pulse dexamethasone, and rituximab (4 weekly doses 375 mg/m^2^). TPO levels were measured serially for a period of 6 weeks (Figure 1E): TPO levels were undetectable during periods of extreme thrombocytosis and increased when platelet counts were low.

Treatment with the oral TPO receptor agonist eltrombopag was started and timed with anticipated periods of thrombocytopenia; specifically, treatment was withheld during anticipated periods of escalating platelet counts and restarted when platelet count was anticipated to drop below 100 × 10^9^/L. The patient received 50 mg of eltrombopag daily from 30 November to 6 December 2010, 2 January to 15 January 2011, and 22 February to 14 March 2011. This resulted in extreme thrombocytosis and did not alter the cycle pattern or period (Figure 1F, periods of treatment within the double arrows); thus, eltrombopag was discontinued thereafter.

### 2.2 Patient 2

**Figure 2:**
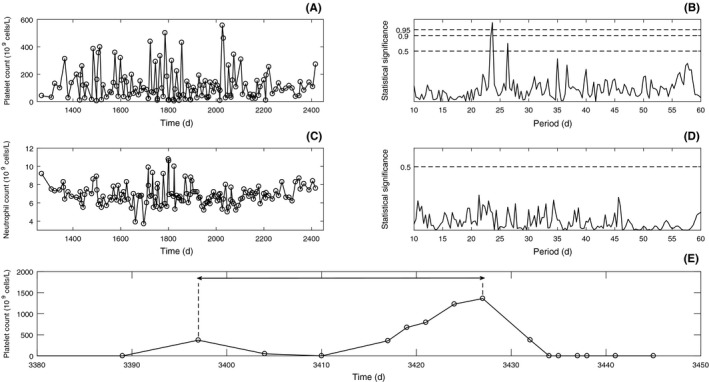
Patient 2 data, with time measured in days since diagnosis. A, Platelet counts (data shown is from April 2003 to April 2006). B, Platelet power spectrum. C, Neutrophil counts (data shown is from April 2003 to April 2006). D, Neutrophil power spectrum. E, Platelet count before and after daily treatment with 50 mg of eltrombopag (data shown is from 5 January 2009 to 2 March 2009, with period of treatment demarcated by dashed lines and double arrows)

A 53‐year‐old male presented with severe thrombocytopenia in 1999. Splenectomy was carried out in October 1999, but he continued to have severe thrombocytopenia and required frequent doses of intravenous immunoglobulin (IVIG) and corticosteroids. Two years later, he was started on a combination of immunosuppressant medications, which included azathioprine, cyclosporine, and mycophenolate for presumed ITP.^8^ In April 2003, the platelet count levels began to oscillate periodically (Figure 2A) with a statistically significant (*P* ≤ .05) period of 23 days (Figure 2B) from a nadir of <10 × 10^9^/L and a peak of 300‐400 × 10^9^/L. During episodes of severe thrombocytopenia, he frequently had bleeding with oral mucosal blood blisters. Treatment with danazol was added but had no effect. The patient's neutrophil count was normal, and despite apparent fluctuations (Figure 2C), statistically significant cyclicity in the neutrophil count was not detected (Figure 2D).

Six years later, eltrombopag was started at a dosage of 50 mg per day. This resulted in extreme thrombocytosis (peak platelet counts 1361 × 10^9^/L) and eltrombopag and all immunosuppressant medications were stopped (Figure 2E, period of treatment within the double arrows). Following that, the patient experienced a period of severe thrombocytopenia (platelets <10 × 10^9^/L) for approximately 4 weeks. Subsequently, eltrombopag was slowly restarted and immunosuppressant medications were re‐introduced. The cyclical thrombocytopenia became less severe with higher nadir platelet values and 4 years later the cyclical pattern resolved. At the last follow‐up in May 2015, the patient's medications were eltrombopag 75 mg daily, and low doses of azathioprine and mycophenolate.

## 3 STATISTICAL EVALUATION OF CYCLICAL COUNTS

We tested the platelet, neutrophil, monocyte, hemoglobin, basophil, and eosinophil counts for statistically significant periodicity in patient 1 using Lomb‐Scargle periodogram analysis.^4^, ^5^ Cell counts and power spectra are shown in Figure 1 for platelets (panels A and B) and neutrophils (panels C and D), with data shown and restricted over a three‐year period (April 2003 to April 2006). The analysis indicates statistically significant cycling at a period of 39 days in both platelets (*P* ≤ 10^−22^) and neutrophils (*P* ≤ .001). Monocytes also showed cycling with the same period (data not shown). There was no evidence for any statistically significant cycling of basophils or eosinophils, and no apparent cycling in erythrocytes based on hemoglobin levels. The cycling of neutrophils was closely entrained with the platelet counts, with the peak of neutrophils preceding the peak of platelets by 8.3 ± 2.0 days. We sequenced the elastase gene for mutations previously reported in cyclic neutropenia^9^; no mutations were found.

## 4 DISCUSSION

Cyclic thrombocytopenia (CT) is a hematological disorder that has serious implications for bleeding and thrombosis risk for patients. It is difficult to treat as conventional ITP treatments are rarely successful. In our two cases, we applied statistical analysis to characterize the periodicity of platelet count fluctuations and report our experience using TPO receptor agonists to treat this condition. We found that platelet count fluctuations are regular and predictable, and that one patient had concomitant, statistically significant cycling in neutrophils. In our patients, we found it difficult to use TPO receptor agonists because they developed extreme thrombocytosis, raising concerns about the risk of thrombosis. A previous report demonstrated the successful use of romiplostim (a TPO receptor agonist medication) for 2 patients: For one patient, peak platelet counts were significantly lower than the patients in our series; and for the second patient, the dose of romiplostim had to be carefully adjusted to avoid extreme thrombocytosis.^3^

Patient 1 had features of classic CT, but with associated cyclical neutropenia. The synchronous cycling of neutrophils with close entrainment of the two cycles suggests a dysregulation involving a common hematopoietic progenitor cell and a shared signaling pathway. The initial response to corticosteroids suggests an autoimmune basis to the original presentation; however, the immune target may be early progenitor cells in the bone marrow affecting precursors of megakaryocytes and granulocytes, rather than platelets in circulation. The synchrony of the peaks suggests that TPO may be driving the process. This is consistent with our observations of fluctuating TPO levels in this patient, as has been shown in other cases.^10^ TPO receptors appear on hematopoietic stem cells, which may explain how both platelets and neutrophils were affected. Platelet cycling has previously been reported in patients with predominant cyclic neutropenia, which may be due to periodic interruptions of neutrophil production and fluctuations of endogenous granulocyte colony‐stimulating factor (G‐CSF) and other cytokines.^11^ Similarly, concomitant cycling of neutrophils has been previously claimed in a patient with pronounced CT,^12^ but when we tested the neutrophil data of that patient we found no evidence of statistically significant oscillations. A preliminary report^13^ of gene expression profile of two male patients with CT published in abstract form noted cyclic changes in interferon responsive genes as well as transcription factors controlling lineage differentiation. Cytokine oscillations have also been documented in periodic hematological diseases, including CT.^10^, ^14^, ^15^, ^16^

Cyclic neutropenia is usually an inherited genetic disorder with onset in early childhood and associated with failure to maintain the myeloid progenitor compartment. Mutations in the gene for neutrophil elastase cause production of an abnormal enzyme that is probably misfolded, thereby triggering apoptosis in the progenitor cells causing severe neutropenia. The rapid recovery of neutrophils is likely due to a surge in G‐CSF and other cytokines in response to the neutropenia. Patient 1 did not have the characteristic features of cyclic neutropenia or mutations in ELANE gene characteristic of the heritable form of this disease.

Thrombopoietin receptor agonists are a recent class of medications used to treat patients with chronic ITP. Patients 1 and 2 in our series were treated with these agents. Patient 1 developed extreme thrombocytosis after treatment with eltrombopag even when doses were timed to coincide with anticipated periods of thrombocytopenia. Patient 2 also developed extreme thrombocytosis after receiving eltrombopag, followed by severe thrombocytopenia once the eltrombopag was stopped. Once eltrombopag and immunosuppressant medications were re‐introduced gradually, the platelet count nadirs improved. These agents must be used with caution in patients with cyclical thrombocytopenia as they can cause extreme thrombocytosis. This occurrence can lead to the abrupt discontinuation of the medication, which will exacerbate rebound thrombocytopenia.^17^

In the past, it was thought that in cyclical thrombocytopenia the fluctuations appear only in the platelets and not in the white or red blood cells. There have been two relatively extensive surveys of the literature on cyclical thrombocytopenia^7^, ^10^ that analyzed well‐documented cases of platelet fluctuations. In no case was there a report of fluctuations in the red or white blood cells. In other existing cyclical hematological disorders like cyclical neutropenia^18^ or periodic chronic myelogenous leukemia,^19^ fluctuations can appear in all major blood cell lines and are all at the same period in each subject. These diseases are believed to arise from the interaction between the hematopoietic stem cell compartment and peripheral control mechanisms. However, because in CT fluctuations had been observed only in the platelets, a destabilization of a peripheral control mechanism was postulated to play a significant role in the genesis of this disorder.^11^, ^20^ Although the control of neutrophil production and the regulation of erythropoiesis have been the subject of numerous modeling studies, there have been fewer treating the regulation of platelets production. One of the earliest was that of Wichmann et al^21^ which was followed by an exposition of their complete model for hematopoiesis.^22^ Von Schulthess et al,^23^ noting the existence of oscillations in the platelet counts of normal humans, put forward a conceptually different model of thrombopoiesis, which was followed by Bélair and Mackey.^24^ Building on this work, Santillan et al^20^ refined the model attempting to understand cyclical thrombocytopenia, and this was subsequently modified by Apostu and Mackey^10^ and Langlois et al,^25^ again to understand the origins of cyclical thrombocytopenia. The latter modeling work has suggested that a defective TPO‐TPO receptor interaction may contribute to the pathogenesis. Recently, Zhang et al^26^ described an interesting case of CT due to a heterozygous loss‐of‐function mutation of TPO receptor, further suggesting that CT may be due to abnormalities with the TPO receptor. It is fair to say, however, that these previous modeling attempts to understand the mechanistic origins of cyclical thrombocytopenia are not capable of fully explaining the observations of statistically significant oscillations with identical periods in both platelets and neutrophils of patient 1.

Our case series is unique because we applied statistical analysis to a large amount of patient data to describe the periods of platelet and neutrophil oscillations, found that one patient had statistically significant concomitant cycling in neutrophils with the same period as in the platelets, a finding that to our knowledge has never been reported in a case of CT, and because we discussed the hazards of using TPO receptor agonists to treat this CT. Neutrophil fluctuations may be underreported because fluctuations in neutrophil count levels do not give rise to symptoms, and because neutrophil levels, even at the nadir, may remain within the normal range.

## ETHICS APPROVAL AND CONSENT TO PARTICIPATE

The study was approved by the Hamilton Integrated Research Ethics Board (REB‐Number: 10‐499‐C). Research ethics board approval forms are available on request.

## CONSENT FOR PUBLICATION

We did not report any personal information and all data were anonymized. A retrospective chart review with ethics approval from the Hamilton Integrated Research Ethics Board is available upon reasonable request.

## CONFLICT OF INTEREST

None declared.

## AUTHORSHIP

GPL: collected a portion of the patient data, contributed to the Lomb‐Scargle periodogram analysis of the patient data, prepared the figures, interpreted the results of the Lomb‐Scargle periodogram, wrote and edited some of the text, and prepared the final version of the manuscript. DMA: contributed the patient data, supervised the chart review, interpreted the results, edited the text, and approved the final version of the manuscript. JP: initiated the collaboration of this project by contacting Michael C. Mackey, initiated data collection, identified patient treatment through reviewing clinical records, initiated patient testing cyclic neutropenia (ELANE Analysis), and was one of the two attending doctors to the patient. BL: interpreted the results, wrote and edited some of the text, and was one of the two attending doctors to the patient. DCD: interpreted the results, was responsible for the ELANE analysis for cyclical neutropenia, wrote, and edited some the text. MCM: initiated the collaboration at the request of J. Potts, contributed to the Lomb‐Scargle periodogram analysis of the patient data, wrote and edited some of the text, and approved the final version of the manuscript.
